# A Systematic Review of Surgical Management Strategies in the Treatment of Peritoneal Carcinomatosis of Neuroendocrine Origin

**DOI:** 10.3390/curroncol30070466

**Published:** 2023-07-01

**Authors:** Megan Fallows, Ambareesh Samant, Harry Wilson, Reza Mirnezami

**Affiliations:** 1 Division of Medicine, University College London, London WC1E 6BT, UK; zchamf0@ucl.ac.uk; 2 Department of Colorectal Surgery, The Royal Free Hospital, London NW3 2QG, UK; a.samant@nhs.net (A.S.); harry.wilson3@nhs.net (H.W.)

**Keywords:** peritoneal neoplasms, carcinomatosis, neuroendocrine tumours (NET), peritoneum

## Abstract

Cytoreductive surgery (CRS) represents the cornerstone of surgical management for peritoneal carcinomatosis (PC) and involves peritonectomy procedures aimed at complete peritoneal tumour resection. Frequently, CRS is combined with hyperthermic intraperitoneal chemotherapy (HIPEC). The combination of CRS + HIPEC is now considered the standard of care in patients with colorectal and ovarian PC. However, the role of this multi-modality treatment approach in patients with PC of neuroendocrine tumour origin (NET-PC) is less well understood. This systematic review provides a summary of available evidence on management strategies for patients with NET-PC. A systematic literature search was performed using Ovid Medline, EMBASE and Cochrane Library databases to identify studies reporting outcomes for patients with NET-PC undergoing surgical treatment. Eligible studies were assessed for methodological quality and design and evaluated for a method of surgical treatment, method of HIPEC delivery, oncological outcomes, and treatment-related morbidity. Eight studies, including a total of 1240 patients with NET-PC, met predefined inclusion criteria and have been included in this review. In three of the included studies, CRS alone was performed for patients with NET-PC, while five studies reported outcomes with combined treatment using CRS plus HIPEC. All studies were performed at tertiary peritoneal malignancy centres. Only one study directly compared outcomes in patients with NET-PC undergoing CRS plus HIPEC compared with CRS in isolation, with no significant difference in overall survival reported. Carefully selected patients with NET-PC may benefit from aggressive surgical treatment in the form of CRS +/− HIPEC. These procedures are best undertaken at centres with expertise in the management of both neuroendocrine tumours and peritoneal malignancy, as both are conditions that require tertiary-level care. The additional benefit of the HIPEC component in this group of patients remains unclear and warrants further investigation in clinical trials. Overall, the quality of data on this subject is restricted by the low number of studies and the variability in treatment methods employed. A multi-national data registry for patients with NET-PC may offer the opportunity to better define treatment algorithms. Translational research efforts in parallel should focus on developing a better biological understanding of NET-PC, with a view to identifying more effective intraperitoneal cytocidal agents.

## 1. Introduction

Gastrointestinal and gynaecological malignancies have the potential to disseminate to the peritoneal surface. The development of peritoneal carcinomatosis (PC) has been shown to result in significantly abbreviated survival and poor quality of life (QOL) [1]. In recent decades, treatment strategies for PC have evolved considerably, and in select cases, cytoreductive surgery, with or without hyperthermic or normothermic intraperitoneal chemotherapy (HIPEC/NIPEC), have demonstrated superior long-term oncological and QOL outcomes, compared with systemic chemotherapy alone. Evidence in the medical literature pertaining to surgical treatment and outcomes for PC is primarily based on data from patients with primary peritoneal carcinomatosis, pseudomyxoma peritonei, PC from gastric cancer, PC from ovarian cancer and PC from colorectal cancer [2,3,4,5]. Peritoneal carcinomatosis of neuroendocrine tumour (NET) origin (NET-PC) is believed to occur in around 20% of patients with primary mid-gut NETs [6]. Regions at particular risk of NET-PC are the peritoneal linings of the pelvic cavity, the pelvic side walls, and the sigmoid colon, arising as so-called “drop metastases”. In addition, the diaphragm, parietal peritoneum, omentum, small bowel mesentery and colonic mesentery are also frequent sites of NET-PC. These patients have been shown to have poorer overall survival than those patients who do not develop PC, even in the presence of other sites of metastatic disease. There is no optimal surgical or medical treatment for NET-PC; however, long-term survival has been demonstrated in select patients undergoing radical intent surgery for NET-PC.

Currently, there are no firm international guidelines to indicate what optimal treatment for NET-PC should be defined as, unlike for ovarian and colorectal PC, where management guidelines are more widely accepted [5]. From a technical perspective, the concept of CRS for NET-PC has been slow to gain traction within peritoneal malignancy centres, as the dense fibrotic reaction that these tumours frequently induce presents unique challenges for peritonectomy. Equally, from the HIPEC perspective, NETs are generally chemo-insensitive, and so there have been concerns that the application of HIPEC may expose the patient to unnecessary morbidity without any oncological advantage being conferred.

In view of the limited available data focusing specifically on NET-PC, the present systematic review was undertaken to summarise all currently available data on treatment strategies and outcomes for patients with NET-PC undergoing CRS +/− HIPEC.

## 2. Materials and Methods

### 2.1. Search Strategy and Study Selection Process

**Figure 1:**
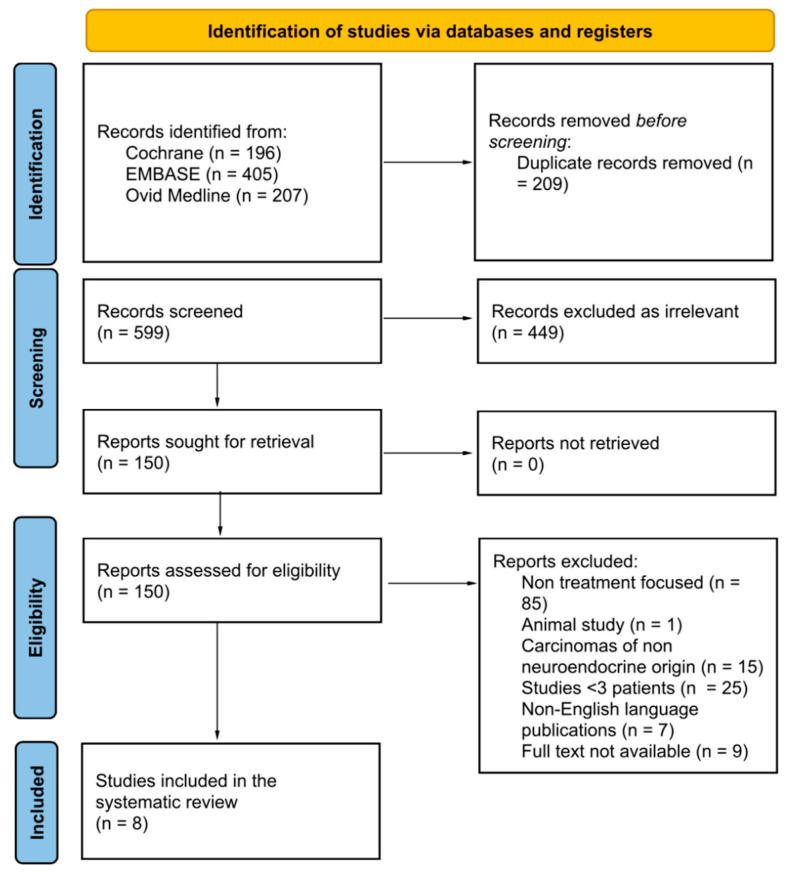
Preferred reporting items for systematic reviews and Meta-Analyses (Prisma) flowchart process.

This systematic review was performed in accordance with the Preferred Reporting Items for Systematic Reviews and Meta-Analysis (PRISMA) guidelines [7]. All resources were last searched in October 2022, and hence all available studies were searched. A thorough literature search was performed in Ovid Medline, EMBASE and the Cochrane Library. To ensure a comprehensive search of the database was carried out, variations of the keywords were used in order to view the full scope of available literature. The following subheadings were combined in the database search for peritoneal carcinomatosis: (‘periton* adj3 (‘cancer’ or ‘carcinoma*’ or ‘neoplasm’ or ‘adenocarcin*’ or ‘tumor’ or ‘tumour’ or amalignan*’)). Furthermore, the following subheadings were combined in the database search for neuroendocrine tumours: (‘neuroendocrine’ adj3 (‘cancer*’ or ‘carcinoma*’ or ‘neoplasm’ or ‘adenocarcin*’ or ‘tumor’ or ‘tumour’ or amalignan*’ or ‘NET’)). Findings were cross-referenced between two researchers, and any discrepancies between findings were discussed and resolved to ensure unanimity. Our systematic review included retrospective and prospective human studies that compared current management strategies in the treatment of NET-PC and evaluated their performance and potential areas for development. The search included eligible studies independent of gender, age, ethnicity, or race. Our search focused on studies reporting data pertaining to NET-PC (including epidemiological data, treatment approaches and treatment-related outcomes). Studies were excluded based on the following criteria: (I) non-available full texts, (II) animal studies, (III) non-English language publications, (IV) studies with <3 patients, (V) studies reporting data on PC of non-neuroendocrine origin, (VI) studies reporting NET-PC but with no mention of treatment. The search algorithm used is summarised in Figure 1. The devised systematic review protocol with registration number CRD42022368499 was approved by PROSPERO on 7 November 2022.

### 2.2. Data Extraction

A data extraction sheet was formulated with extracted variables, including accession numbers, authors, year of publication, journal, country, title, study type, number of patients with NET-PC, symptoms profile associated with treatment, treatment methods used for peritoneal metastases, outcomes and treatment-related morbidity or mortality. Any discrepancies in findings in the table were discussed between researchers (MF and RM) and resolved through discussion.

### 2.3. Risk of Bias Assessment

The risk of bias was assessed using the risk of bias in the non-randomised studies of interventions (ROBINS-I) tool, accessed via the Cochrane database [8]. This step was necessary as the studies were primarily composed of prospective and retrospective case series. The seven bias domains analysed by the ROBINS-I tool are (I) bias due to confounding, (II) bias in the selection of participants into the study, (III) bias in the classification of interventions, (IV) bias due to deviations from intended interventions, (V) bias due to missing data, (VI) bias in the measurement of the outcome, (VII) bias in the selection of the reported result. This assessment has been included in the Supplementary Materials Section.

## 3. Results

### 3.1. Literature Search and Study Characteristics

A total of 808 studies were identified in the literature, with 599 remaining after the removal of duplicate records. Following the screening process, eight studies remained, which were included in this review [9,10,11,12,13,14,15,16]. Of the eight studies, four were conducted in France [9,13,15,16], one in the USA [14], one in Denmark [11], one in Germany [12] and one was conducted in Canada [10]. Table 1 highlights the study and population characteristics in full, with all primary tumour locations and the number of patients in each individual study.

### 3.2. Study Population

A total of 1240 patients were included and analysed in this study. All eight studies included patients being treated with CRS, and five studies also investigated the use of HIPEC as an adjunct strategy alongside CRS [11,12,13,15,16]. Table 2 provides a more in-depth analysis of how the peritoneal disease was assessed prior to surgery, whether the peritoneal disease was synchronous or metachronous, and which patients had synchronous peritonectomy with/without HIPEC in each study. Table 3 provides a summary of the methods of treatment administration, including temperature, chemotherapeutic agents, and adjunct treatments. HIPEC administration was intraoperative in all studies in which HIPEC was used as an adjunct treatment. The chemotherapeutic agents used for HIPEC varied greatly, as mentioned in the table, but two of the five HIPEC studies used 5-fluorouracil as an agent [11,15]. The temperature of the HIPEC administration ranged from approximately 40–43 °C in the included studies.

### 3.3. Treatment Associated Morbidity/Mortality

CRS was performed in all eight studies and is currently the main treatment for NET-PC patients [11]. The quality of the CRS was assessed in most studies [9,12,13,15,16] using the ‘Sugarbaker Completeness of Cytoreduction Score’ [13].

Benhaim et al. reported two cases of early post-operative mortality following CRS + HIPEC. One was in the LM group, and the other was in the LM + PM group [9]. Chan et al. further reported two cases of mortality after CRS + HIPEC in the post-operative period [10]. Of the 14 patients studied by Brandl et al., eight were treated with postsurgical HIPEC which lasted 60 min. Non-surgical complications included 1 cardiac, 3 gastrointestinal, 4 infectious and 5 respiratory disorders [12]. Whereas Madsen et al. highlighted post-operative complications during the immediate hospital stay of 8 patients, including 3 pneumonia, 2 urinary tract infections, 3 fever, 2 anastomotic leakage and 1 wound dehiscence [11]. Goere et al. followed patients where a combined CRS and HIPEC treatment plan was implemented and noted post-operative complications in 272 of the 781 patients with NET-PC of Clavien-Dindo grade III-IV severity [13]. Woltering et al. reported intraoperative complications in 94 of the 1001 procedures performed, these were ranked as major or minor post-operative complications and varied from complications such as those involving the lung, small bowel, and pancreas [14]. Elias et al. reported that multi-modality treatment with CRS + HIPEC was the main factor responsible for increased intraoperative blood loss, and that this treatment was independently a more significant factor related to blood loss, even when compared with extent of hepatic resection, in patients undergoing concurrent liver metastasectomy [15]. Importantly, the authors found that the incidence of post-operative complications, were similar amongst patients in the CRS group versus patients undergoing CRS + HIPEC. The authors postulated that the CRS component of treatment may be the main driver of morbidity in these patients. On the other hand, it is interesting to note that in a more recent study, Hajjar and colleagues, reported significantly inferior morbidity outcomes in patients undergoing CRS + HIPEC compared with CRS alone [16]. Here, the authors reported a 50% risk of grade III-IV morbidity in the CRS + HIPEC group (16 out of 32 patients), compared to just 3.4% in the CRS alone cohort (1 out of 29 patients). Furthermore, one patient died during the post-operative period, who belonged to the combined CRS + HIPEC group.

Table 4 summarises the respective morbidity and mortality rates and overall survival.

### 3.4. Survival Outcomes

Treatment associated survival outcomes were provided by all eight of the studies included in this review, and these are summarised in Table 4. It is challenging to draw direct comparisons in terms of these outcomes, due to study heterogeneity, with varying treatment algorithms, widely varying patient populations and variable reporting of survival. Five-year overall survival was reported in five of the eight studies and ranged from 39.9% [13] to 91.6% [16]. It is perhaps somewhat surprising to see such wide variation in survival outcomes, though this is likely to be the result of significant between study heterogeneity, and variability in case selection. Hajjar and colleagues interestingly reported significantly inferior 5-year overall survival in patients undergoing CRS + HIPEC, compared with CRS alone (74.5% versus 91.6%, respectively). In addition, it is important to note that although Hajjar et al. reported recurrence free survival of 0% in the CRS alone group of patients, these patients still achieved a remarkable overall survival of 91.6%. This finding would imply that patients with NET-PC treated with radical intent, have a high likelihood of succumbing to disease relapse, and yet this does not necessarily result in truncated overall survivorship. Furthermore, Hajjar et al. demonstrated a trend towards improved disease-free and recurrence-free survival, respectively, in patients undergoing CRS + HIPEC, compared with CRS alone (Table 4).

## 4. Discussion

Peritoneal carcinomatosis of neuroendocrine origin affects around 20% of patients with primary mid-gut NETs and imparts a significant negative impact on quality of life and cancer survivorship. The fundamentally diffuse nature of PC combined varying treatment strategies makes homogenisation of data across different study cohorts challenging. As well as data heterogeneity, there is the additional problem of limited patient numbers, as evidenced in this review, where the combined patient population was just 1240. On the basis of these inherent limitations, defining the optimal surgical treatment strategy for patients with NET-PC has proved challenging. The North American Neuroendocrine Tumour Society (NANETS) guidelines published in 2020 acknowledge the unique challenges that NET-PC pose to the surgical oncologist, and equally acknowledge the considerable QOL impact experienced by these patients. This guidance advocates surgical excision of all visible and resectable disease, whilst at the same time minimising treatment associated harm to the patient and furthermore, avoiding excessively radical resection [17]. This guidance is somewhat nebulous, likely a reflection of the data limitations highlighted. These shortcomings notwithstanding, the present review was undertaken in order to provide the first comprehensive summary of data on this subject, with a view to developing more well-defined treatment recommendations in the future.

In terms of demonstrating the efficacy of CRS for patients with NET-PC, Elias and colleagues were the first to report an ostensible 17–25% improvement in survival of patients treated with CRS at their tertiary peritoneal malignancy centre, compared with survival outcomes achieved with conventional treatment [15]. Since then, there has been a growing interest in the application of CRS in the treatment of NET-PC. The application of HIPEC in this context has remained controversial.

The use of CRS in the management of NET-PC appears to show good efficacy in terms of survival benefit. Benhaim et al. demonstrated that patients who had NET with PC alone had the best 5-year survival prognosis (81%), compared to patients with liver metastasis (78%) or both liver and peritoneal metastasis (72%) [9]. Woltering also demonstrated similar 5-year survival rates when CRS was used in isolation with 82% at 5 years [13].

The study by Madsen et al. is the only one where HIPEC was used prophylactically in a cohort of patients deemed to be at high risk of PC and this cohort had a 5-year overall survival rate of 100% [11]. The high-risk features were classified as having at least one of the following: perforated appendiceal tumour, periappendicular abscess and resection margin <1 mm.

Only two studies have been identified that include comparative data for patients with NET-PC undergoing CRS alone versus CRS + HIPEC. Elias et al. demonstrated that both cohorts have similar one year survival figures with a one-year survival of 88% in the CRS group and 89% in the CRS + HIPEC group [15]. The CRS + HIPEC group demonstrated improvements in disease-free survival of 77% compared to 49%, which may evidence the capability of HIPEC to abolish microscopic residual peritoneal metastases. Hajjar et al., however, reported inferior 5-year survival in the cohort of patients undergoing CRS + HIPEC, versus CRS in isolation [16]. The 5-year survival in the CRS alone group was 91.6% compared to 74.5% in the combined group. However, this observation may be down to selection bias, with patients with higher PCI scores being selected for more aggressive combined-modality treatment. Additionally, the 5-year survival of patients in this study undergoing CRS alone patients appears to be higher than previous studies, highlighting a possibility of said selection bias for patients with a reduced peritoneal disease burden. Hajjar et al. demonstrated an improvement in recurrence free survival (0% in CRS group versus 30.8% in CRS + HIPEC group), which again may highlight the ability of HIPEC to reduce the risk of recurrence from micro metastases on the peritoneal surface [16].

The studies presented in this review demonstrate an increased incidence of post-operative complications when HIPEC is used alongside CRS for NET-PC. Elias et al. reported an increase in the volume of blood loss when HIPEC was added [15]. Alongside this, a substantial increase in major post-operative morbidity was noted when HIPEC was added, occurring in 61% of patients versus 39% in the non-HIPEC group. Goere et al. recorded a high volume of grade III-IV adverse events in 272/781 (34.8%) patients that received CRS + HIPEC though they did not have a control group for comparison [13]. Hajjar et al. similarly reported a significantly higher proportion of grade III-IV adverse events after surgery in patients undergoing CRS + HIPEC compared with CRS alone (50% versus 3.4%, respectively).

The present study is subject to several inherent limitations, which the authors wish to acknowledge. Firstly, the volume of data presented is low, comprising 1240 patients from eight different clinical studies. Heterogeneous study populations and treatment algorithms are further areas of between-study variability, and to date, no randomised controlled trial has been undertaken to evaluate the impact of CRS with or without HIPEC in NET-PC. These shortcomings are worth highlighting and should serve as a ‘call to arms’ within the NET surgical oncological community to generate higher-quality data in the field. It is also worth appreciating, however, that the data presented in this review offer a contemporary snapshot of real-world experiences to date in a group of patients who are challenging to homogenise. These limitations notwithstanding, the data presented herein indicate that currently, as far as clinical and translational research efforts, the scientific evaluation of optimal surgical treatment of NET-PC remains relatively embryonic. There are conflicting reports regarding local control enhancement with the addition of HIPEC to CRS, and this is at least in part likely to be the result of data heterogeneity. In general, it is acknowledged that well-differentiated NETs respond poorly to conventional systemic chemotherapeutic regimens, and therefore, one could postulate that the same is likely to apply in the case of intra-peritoneally administered agents. A departure from standard HIPEC regimens will require translational research efforts to identify optimal agents for this purpose, specifically targeted towards NET-PC. Simultaneously, and given the relative rarity of these tumours, the authors believe that the instigation of a multi-national data repository evaluating treatment strategies and outcomes for patients with NET-PC may offer the best opportunity to standardise practice, develop novel research avenues and improve patient outcomes.

## 5. Conclusions

Overall, there is currently no level 1 evidence regarding the treatment for NET-PC. Furthermore, there is little comparative data in terms of the oncological impact of CRS on NET-PC; hence, there is minimal data where well-matched patient cohorts are compared for CRS (with or without supplemental HIPEC) versus non-operative treatment. In addition to this, NET-PC is an area with little translational research, and thus, this needs immediate development.

From the studies included in this review, CRS does appear to offer benefits for patients of NET-PC but may be limited to a small subset of patients. There is currently insufficient data to support the routine use of HIPEC as an adjunct to CRS for patients with NET-PC, and future studies are likely to better determine the role of this treatment. In the future, translational studies evaluating the potential for novel intra-peritoneal agents designed specifically for NET-PC should be initiated.

## Figures and Tables

**Table 1:** Summarised findings of the study and population characteristics.

First Author	Year	Country	Study Design	Total Number of Study Patients (N)
Benhaim [9]	2021	France	Prospective	88
Chan [10]	2018	Canada	Prospective	55
Madsen [11]	2018	Denmark	Prospective Cohort	48
Brandl [12]	2017	Germany	Retrospective	14
Goere [13]	2017	France	Retrospective	127
Woltering [14]	2017	USA	Retrospective	800
Elias [15]	2014	France	Prospective/Retrospective	41
Hajjar [16]	2022	France	Prospective	67

**Table 2:** Procedure Characteristics.

First Author	Year	Method of PC Assessment and Staging Prior to CRS +/− HIPEC	Synchronous vs. Metachronous Peritoneal Disease	Synchronous Peritonectomy +/− HIPEC	PCI Criteria for Determination of Resectability
Benhaim [9]	2021	- CgA levels recorded, CT scans, somatostatin scintigraphy, liver MRI - Additionally, evaluated anaestheologist examination with echocardiography and NT-proBNP	- Synchronous	- No HIPEC used	- Mean PCI 10 (PM group), and 11 (PM + LM group) - All PCI levels considered for surgery
Chan [10]	2018	- No specific methods mentioned	- Synchronous	- No HIPEC used	- Not mentioned
Madsen [11]	2018	- PET/CT in all patients, colonoscopy performed to exclude synchronous tumours in large bowel - Evaluated laparoscopically if eligible for HIPEC	- Synchronous	- Synchronous	- Excluded if had PC in 6 or more regions according to Dutch 7 region count score
Brandl [12]	2017	- CT scans of chest, abdomen and pelvis and tumour markers (CA 19-9, CEA, CA 125) obtained	- Synchronous	- Synchronous-HIPEC administered immediately after cytoreduction	- Not mentioned as an exclusion criterion but was used to assess PM extent
Goere [13]	2017	- No specific methods mentioned	- Combination	- HIPEC used	- Extent of PC assessed using PCI and median score was 12 - Patients with incomplete cytoreduction were excluded from the disease-free survival analysis
Woltering [14]	2017	- Pathology reports reviewed to ensure all NETs	- Synchronous	- No HIPEC used	- Not mentioned as exclusion criteria
Elias [15]	2014	- Abdominal, pelvic, thoracic CT, ultrasonography, MRI, bone scintigraphy, somatostatin receptor scintigraphy - Sugarbaker CCRS	- Synchronous	- Period of 1994–2007 CCRS was combined with HIPEC - Period of 2008–2012 CCRS performed without HIPEC	- KI-67 index NOT used - PCI median 14 (HIPEC group), 11 (non-HIPEC group)
Hajjar [16]	2022	- PM burden assessed using PCI - Residual disease after CRS assessed using CCRS	- Synchronous	- One group just CRS and the other CRS + HIPEC	- Not mentioned as an exclusion criterion but was used to assess PM extent

**Table 3:** Summary of treatment characteristics.

Figure	Year	Treatment	Surgical Treatment	HIPEC Strategy	Post-Operative Adjuvant Therapy
Benhaim [9]	2021	CRS	Surgical resection for grade 1 or 2 tumour patients. Resection performed using peritonectomy and organ resections. Liver clearance used to decide surgical resection or radiologic chemoembolisation performed prior to surgical resection	N/A	N/A
Chan [10]	2018	CRS	All surgeries performed through laparotomy by 2 NET specialised surgeons. Extrahepatic and hepatic metastases (parenchyma sparing enucleation) are respected. ECG and octreotide preparation before surgery if they had elevated 24-hr urinary 5-HIAA.	N/A	N/A
Madsen [11]	2018	**Group 1**—Right-sided hemicolectomy—patients with GCC limited to non-perforated appendix **Group 2**—CRS + prophylactic HIPEC-patients with GCC and high risk of carcinomatosis (+perioperative chemotherapy if feasible) **Group 3**—CRS + HIPEC-patients with peritoneal carcinomatosis **Group 4**—Systemic palliative chemotherapy-patients with extensive carcinomatosis	Group 1—right-sided hemicolectomy performed laparoscopically. Explorative laparotomy performed to decide if a patient should have HIPEC + CRS. If CRS + HIPEC criteria were not met when surgery began, an open-closed procedure was completed or palliative surgery (chosen by surgeon)	If eligible for CRS + HIPEC, the patient received neoadjuvant chemotherapy with 5-fluorouracil or capecitabine and oxaliplatin. After CRS, abdomen perfused with peritoneal dialysis solution added mitomycin C 35 mg/m^2^ at 41.0–42.5 °C for 90 min.	For 6 months after treatment, patients offered adjuvant systemic chemotherapy with CapOx or FolFox.
Brandl [12]	2017	CRS, HIPEC	Complete cytoreduction defined as nodules less than 2.5 mm in size (CC = 1) or absence of visible nodules (CC = 0)	HIPEC protocols differed depending on tumour properties. 83.3% of cases, HIPEC delivered for 60 min and via a closed abdomen technique in 71.4% of patients, with open circulation used in the remaining patients. The mean temperature of the chemoperfusion was 40.9 °C	N/A
Goere [13]	2017	CRS + HIPEC	Quality defined according to the ‘Sugarbaker completeness of cytoreduction score’ (CC) Cytoreductive surgery was considered to be complete when a CC-0 or CC-1 score was attained.	Intraperitoneal treatment consisted of HIPEC either done with the open abdomen or the closed abdomen technique. Median duration of HIPEC 90 min (25–180) and the mean temperature of the bath was 41.9 °C. Chemotherapeutic agents used were cisplatin, doxorubicin, mitomycin C, oxaliplatin and irinotecan	N/A
Woltering [14]	2017	CRS	73% of patients treated with SSA therapy before undergoing CRS 57% of patients had resection of the primary tumour with simultaneous resection of hepatic and/or extrahepatic metastases only 41 operations were limited to just removing the primary tumour	Additional preoperative treatments-chemotherapy, yttrium-90 microsphere embolisation, bland embolisation, transarterial chemoembolisation, peptide receptor radionuclide therapy, external beam radiation therapy (XRT) and radioactive iodine-131 metaiodobenzylguanidine therapy	N/A
Elias [15]	2014	CRS + immediate HIPEC (28) CRS alone (13)	CRS defined as resection of all tumour deposits greater than 1 mm in diameter	The HIPEC protocol included intravenous infusion of 20 mg/m^2^ of leucovorin followed by 400 mg/m^2^ of 5-fluorouracil. Then, HIPEC was performed over 30 min at an intraperitoneal temperature of 43 °C, with oxaliplatin alone or mixed with irinotecan, in 2 L/m^2^ of 5% dextrose. The doses of oxaliplatin were 460 mg/m^2^ when given alone but 300 mg/m^2^ when mixed with irinotecan (200 mg/m^2^).	N/A
Hajjar [16]	2022	CRS + HIPEC (36) CRS alone (31)	Combined treatment group-open technique performed in 77.1% and closed technique in 22.9%. Median operation time for the combined group was 8.1 h, compared to 5.9 h in the CRS alone group.	Not Specified	N/A

**Table 4:** Summary of mortality, morbidity, and overall survival.

First Author	Year	Morbidity/Mortality	Survival Data
Benhaim [9]	2021	- 2 patients (out of 88) died from post-operative complications	5 year overall survival rates: - 81% in peritoneal group - 78% in liver group - 72% in the peritoneal + liver group
Chan [10]	2018	- 2 patients (out of 55) died within 30 days of the surgery	5 year overall survival rates: - 81.5% for whole cohort
Madsen [11]	2018	- 8 patients (out of 48) developed post-operative complications during hospital stay	5 year survival rate: - 100% in Groups 1 and 2 - Group 3 median survival time was 3.2 years - Group 4 median survival time was 1.3 years
Brandl [12]	2017	- Post-operative morbidity 50% (out of 14 patients)	- Overall survival post 16 month follow-up—43%
Goere [13]	2017	- 19 patients (out of 127) died post-operatively	- 5 year survival rate—39.9%
Woltering [14]	2017	- 229 patients (out of 800) died during study	- 5 year overall survival rate—82%
Elias [15]	2014	- 1 patient died (out of 41) post-operativelyGrade III-IV morbidity was 56%	- 5 year overall survival rate—69%
Hajjar [16]	2022	- Grade III-IV morbidity experienced in 50% of the combined group compared to 3.4% in CRS alone group (out of 67 patients)	- 5 year overall survival rates: - 91.6% (CRS alone) - 74.5% (CRS + HIPEC)

## Supplementary Materials

The following supporting information can be downloaded at: https://www.mdpi.com/article/10.3390/curroncol30070466/s1, Table S1: Risk of Bias Domains.
