# Supplementary material for: A Systematic Review of Surgical Management Strategies in the Treatment of Peritoneal Carcinomatosis of Neuroendocrine Origin
Source: Curr Oncol. 2023 Jul 1;30(7):6316–29. doi: 10.3390/curroncol30070466 (PMC10378295; doi:10.3390/curroncol30070466)
Supplement: Supplementary file 1 [file curroncol-30-00466-s001.zip › curroncol-2387237-Supplementary Materials.pdf]

Supplementary Materials:

Quality Assessment:

The ROBINS-I tool was used to assess the risk of bias in each of the individual studies. From this, the overall risk of bias was deemed to be low in three studies [7,10,14], moderate in three studies [8,11,13], and serious in two studies [9,12]. A summary of the risk of individual biases are highlighted in Table S1.

Table S1. Risk of Bias Domains.

Study	D1	D2	D3	D4	D5	D6	D7	Overall
Benhaim 2021	+	+	+	+	+	+	+	+
Chan 2018	−	+	+	−	+	+	+	−
Madsen 2018	−	−	+	X	+	+	+	X
Brandl 2017	+	+	+	+	+	+	+	+
Goere 2017	+	−	+	−	−	+	+	−
Woltering 2017	+	+	+	X	+	+	+	X
Elias 2014	−	−	+	+	−	+	+	−
Hajjar 2022	+	+	+	+	−	+	+	+

Domains:

D1 - Bias due to confounding
D2 - Bias due to selection of participants
D3 - Bias in classification of interventions
D4 - Bias due to deviations from intended interventions
D5 - Bias due to missing data
D6 - Bias in measurement of outcomes
D7 - Bias in selection of the reported result

Judgement:

X Serious
− Moderate
+ Low
